# CircRNA hsa_circ_0002577 accelerates endometrial cancer progression through activating IGF1R/PI3K/Akt pathway

**DOI:** 10.1186/s13046-020-01679-8

**Published:** 2020-08-26

**Authors:** Yu Wang, Lili Yin, Xiaofei Sun

**Affiliations:** Department of Obstetrics & Gynecology, Shengjing Hospital Affiliated to China Medical University, No. 36 Sanhao Street, Heping District, Shenyang City, 110004 Liaoning Province China

**Keywords:** Circular RNA, Endometrial cancer, IGF1R, miRNA sponge, PI3K/Akt pathway

## Abstract

**Background:**

Endometrial cancer (EC) is a common gynecologic malignancy worldwide. This study investigated the regulatory effects of circular RNA (circRNA) hsa_circ_0002577 on the tumorigenesis of EC.

**Methods:**

Tumor samples and adjacent normal tissues were obtained from 84 EC patients. Recombinant lentiviral vectors expressing hsa_circ_0002577 (Lv-circRNA), short hairpin RNAs against hsa_circ_0002577 (sh-circRNA), miR-625-5p mimics, miR-625-5p inhibitor, lentiviral vectors expressing insulin-like growth factor 1 receptor (IGF1R) and their corresponding controls were transfected into EC cells as designated. A mouse xenograft model was established in BALB/c mice by inoculating Ishikawa cells transfected with sh-circRNA or control sequence.

**Results:**

Hsa_circ_0002577 was upregulated in EC tissue samples and cells as compared to normal controls. EC patients with higher expression of hsa_circ_0002577 showed poorer overall survival and more advanced tumor stage. EC cells transfected with Lv-circRNA showed promoted proliferation, migration, and invasion, whereas the delivery of sh-circRNA exerted an opposite effect. Further analyses showed that hsa_circ_0002577 acted as a miR-625-5p sponge in EC cells. IGF1R was a potential downstream target of miR-625-5p. The expression of IGF1R in EC tissues was significantly higher than that in matched controls. Hsa_circ_0002577 accelerated EC development by inducing IGF1R expression and activating PI3K/Akt signaling pathway. Also, the knockdown of hsa_circ_0002577 delayed tumor growth and metastasis in the inoculated mice.

**Conclusion:**

Our study showed that circRNA hsa_circ_002577 accelerated EC progression by acting as a miR-625-5p sponge, upregulating IGF1R and activating the PI3K/Akt pathway, suggesting the potential therapeutic use of hsa_circ_002577 in EC treatment.

**Trial registration:**

Not Applicable.

## Background

Endometrial cancer (EC) is the fourth commonly diagnosed gynecologic malignancy worldwide with an annual increase in incidence of approximately 1–2% [1, 2]. In China, EC ranks second among female genital cancers with a mortality rate of 2.7 per 100,000 [3]. The International Federation of Gynecology and Obstetrics (FIGO) system is widely used to determine EC stages, which range from I to IV [4]. Seventy-five percent patients are diagnosed at an early stage (I/II) and their overall 5-year survival rate was over 70%. However, the survival rates of women who are diagnosed at FIGO stage III or IV are 57–66% and 20–26%, respectively [5]. The treatment options for EC depend largely on the stage of the disease. Surgeries include pelvic lymph node dissection, bilateral salpingo-oophorectomy, and hysterectomy remain as the mainstay of EC treatment for early diagnosed patients [6]. For patients with distant metastasis or local recurrence, adjuvant radiotherapy, adjuvant chemotherapy, and the use of anti-angiogenic agents are also included [7]. Several molecular targeted therapies for EC are currently under investigation, such as phosphoinositide 3-kinase (PI3K)-AKT-mammalian target of rapamycin inhibitors and epidermal growth factor receptor inhibitor [8, 9]. The identification of novel molecular targets facilitates the development of diagnostic and therapeutic approaches for EC patients.

Circular RNAs (circRNAs) are a class of covalently closed, single-stranded RNAs generated from precursor mRNAs by back-splicing [10]. Growing evidence has shown that circRNAs may contribute to the regulation of various biological processes through inhibiting microRNAs (miRNAs), harboring the binding site for RNA-binding protein, and regulating the transcription, translation, or epigenetic alterations of target genes [11]. The lack of 5′-3′ ends and poly-adenylated tails makes circRNAs insusceptible to the degradation by RNase R, therefore much more stable than linear RNAs [12]. Recently, the potential use of circRNAs as therapeutic targets and biomarkers in human carcinomas has been highlighted [13]. However, whether circRNAs may have regulatory effects on EC development remains unknown. A study of circRNA expression profile in EC patients reported that the serum level of hsa_circ_0002577 in EC patients was 2.4 times higher than that in healthy subjects, while the fold change of other tested circRNAs ranged from 1.43–2.05 [14]. WD repeat domain 26 (WDR26) gene is a precursor of hsa_circ_0002577. It has been reported that WDR26 is upregulated in malignant breast tumors, and the upregulation of WDR26 facilitated the growth and metastasis of breast cancer by stimulating the activation of PI3K/AKT pathway [15]. Chen et al. found that a long-non-coding RNA lncWDR26 suppressed the tumorigenesis and metastasis of hepatocellular carcinoma via repressing the transcription of WDR26 [16]. The above information implied that the aberrant expression of hsa_circ_0002577 in EC might exert detrimental effects on the pathogenesis of the tumor.

In the current study, the expressions of hsa_circ_0002577 in EC tissue samples and cell lines were examined. The regulatory effects of hsa_circ_0002577 on EC progression were explored in both cell and mouse xenograft models. Further investigation revealed that hsa_circ_0002577 acted as a miRNA sponge for miR-625-5p, mediated the expression of its target gene *IGF1R*, and regulated the activation of PI3K/Akt signaling pathway during the development of EC.

## Methods

### Human tissue samples

EC tissues and matched adjacent normal tissues were obtained from 84 patients undergoing hysterectomy for EC at the Shengjing Hospital Affiliated to China Medical University. No patient received radiotherapy or chemotherapy before operation. The histological diagnosis and FIGO staging of EC were performed by two experienced pathologists. The fluorescence in situ hybridization (FISH) was performed to compare the level of hsa_circ_0002577 in EC and normal tissues as previously described [17]. Briefly, paraffin-embedded tissue samples were cut into 5 μm sections. The hybridization mix was prepared with 2 μL of hsa_circ_0002577 probe (0.5 ng/μL) in the 18 μL hybridization buffer containing 25% (v/v) formamide, 2× SSC, 5× Denhardt’s solution, 1 mM ethylenediaminetetraacetic acid, and 50 nM phosphate buffer at 37 °C overnight. After three washes with 2× SSC and one wash with RNase-free water, slides were stained with DAPI. After treating with ProLong® Gold Antifade (Invitrogen, Carlsbad, USA) overnight in the dark, images were captured at 40× and 100× magnification using a fluorescence microscope. All experiment protocols were approved by the Medical Ethics Committee of the Shengjing Hospital Affiliated to China Medical University and performed following the World Medical Association Declaration of Helsinki [18]. Each participant provided written informed consent.

### Cell culture

EC cell lines (HEC-1-B, AN3-CA, KLE, HEC1-A, and Ishikawa) and human endometrial endothelial cells (hEEC) were obtained from ATCC (Shanghai, China) and maintained in DMEM medium supplemented with 10% fetal bovine serum. HEK-293 T cells (ATCC) were cultured in DMEM medium containing 4.5 mg/mL glucose, 2 nM glutamine, 10% fetal calf serum, and 1% streptomycin (Sigma-Aldrich, St. Louis, USA). Human umbilical vein endothelial cells (HUVECs, ATCC) were cultured in Endothelial Cell Growth Media (ECGM, #211–500, Sigma-Aldrich). All cells were maintained in a humidified atmosphere of 5% CO_2_ at 37 °C.

### Cell transfection

Recombinant lentiviral vectors expressing hsa_circ_0002577 (Lv-circRNA), short hairpin RNAs (shRNAs) against hsa_circ_0002577 (sh-circRNA), miR-625-5p mimics, miR-625-5p inhibitor, lentiviral vectors expressing insulin-like growth factor 1 receptor (IGF1R) and their corresponding negative controls (Lv-NC, sh-NC, NC inh, NC miR, Control, respectively) were designed and synthesized by GenePharm (Shanghai, China). When reached 60–70% confluency, EC cells were transfected with designated vectors and/or sequences for 48 h using the TransFast transfection reagent Lipofectamine 2000 (Invitrogen).

### RNase R enrichment

RNase R is a 3′ to 5’exoribonuclease that degrades linear RNAs without affecting circular RNAs [19]. RNase R treatment was performed as previously described to enrich circular RNAs [20]. In brief, RNAs were isolated from Ishikawa and HEC-1-B cells using RNAeasy system and on-column DNAse digestion (Qiagen, Hilden, Germany). In the first replicate, 60 μg total RNAs were depleted for ribosomal RNAs using RiboMinus kit (Invitrogen) in 6 separate 10-μg reactions. In the second biological replicate, 20 μg total RNAs were depleted for ribosomal RNAs. A total of six 14.3-μL RNase R reactions or mock treatment aliquots were prepared for each replicate. Samples were denatured at 70 °C and then chilled to 40 °C using a thermocycler. Next, 1.7 μL 10× RNase R buffer was added to each sample. A volume of 1 μL water was added in one of the six reactions, and 1 μL RNase R was added to the remaining reactions. The reactions proceeded for 1 h at 40 °C.

### Cell proliferation assessments

Cell proliferation was evaluated using CCK-8 assay and colony formation assay. In CCK-8 assay, cells (2.5 × 10^4^ cells/well) were plated into 96-well plates and transfected with designated vectors and/or sequences. At indicated time points (0, 24, 48, 72, and 96 h) post-transfection, cells were incubated with 10 μL CCK-8 solution (Dojindo, Tokyo, Japan) for 3 h at 37 °C. The absorbance value was measured at 450 nm wavelength using a micro-plate reader (Bio-Rad, Hercules, USA). In colony formation assay, cells (1.5 × 10^3^ cells/well) were seeded into P100 petri dishes and transfected with designated vectors and/or sequences for 10 days. Then cells were fixed and stained with 1% crystal violet (Sigma-Aldrich). The quantity of colonies in each petri was counted from 6 randomly selected and normalized to the number of colonies in the control group.

### Apoptosis detection

Following transfection, cells were fixed and treated with DAPI (Thermo Fisher Scientific, Waltham, USA) and TUNEL (Roche, Basel, Switzerland) reagents. DAPI stained cell nucleus. The apoptotic cells were detected by dual DAPI and TUNEL staining. The percentage of TUNEL-positive cells on each slide was calculated in 6 randomly selected fields by using a fluorescence microscope at × 100 magnification.

### Assessments of cell migration/invasion

To assess the migration/invasion capability of cells, wound healing assay and Transwell assay were performed. In would healing assay, cells were plated in 6-well plates and transfected with designated vectors and/or sequences for 48 h. Then an artificial straight scratch was made using a sterile pipette tip. At 0 h and 24 h after scratching, the relative migration rate (%) was determined by measuring the percentage of wound recovery area under a light microscope (magnification, × 100). In Transwell assay, transfected cells were transferred to the upper chamber of 24-well Transwell plates with 8 μm-pore membrane (BD Biosciences, Bedford, USA) and cultured in serum-free medium. The bottom chamber was added with DMEM containing 10% fetal bovine serum. Twenty-four hours later, the invading cells at the bottom chamber were fixed and stained with crystal violet dye. The number of migrated cells was counted in 6 randomly selected fields under a light microscope (magnification, × 100).

### HUVECs tubule formation assay

The cell supernatants were collected from EC cells transfected with designated vectors and/or sequences. HUVECs were cultured with a mixture of EC cell supernatants and ECGM for 4 days. Then HUVECs were trypsinized and reseeded (3 × 10^4^ cells/well) onto Matrigel-coated wells. Twenty-four hours later, the branching points of HUVECs, defined as the three overhanging branches of the intersection point, were observed under a Leica microscope and the branches were quantified using Image-Pro software.

### Immunofluorescence staining of EC cells

EC cells were grown on glass multi-chamber slides (BD Biosciences, San Jose, USA), fixed with 4% paraformaldehyde, and permeabilized with 0.4% Triton X-100 (Sigma-Aldrich). Then slides were treated with a hybridization mixture containing 18 μL hybridization buffer, 1 μL hsa_circ_0002577 probe (0.5 ng/μL) and 1 μL miR-625-5p probe (0.5 ng/μL) at 37 °C overnight. After treatment with RNase-free water and ProLong® Gold Antifade, slides were stained with DAPI and the images were captured using a fluorescence microscope at 200× magnification.

### Dual-luciferase reporter assay

***Experiment 1:*** The 3′-UTR fragment of hsa_circ_0002577 containing the putative binding site of miR-625-5p or a mutant sequence was cloned into pmirGlo vectors (GenePharm). HEC-1-B cells at 70–80% confluence were co-transfected with miR-34b-5p inhibitor (or NC inh) and hsa_circ_0002577-WT (or hsa_circ_0002577-MUT) for 48 h. Ishikawa cells were co-transfected with hsa_circ_0002577-WT (or hsa_circ_0002577-MUT) and miR-34b-5p mimics (or NC miR) when reached 70–80% confluence. ***Experiment 2:*** The 3′-UTR fragment of IGF1R harboring the putative binding site of miR-625-5p or a mutant site was cloned into pmirGlo vectors. The IGF1R-WT (or IGF1R-MUT) and miR-34b-5p inhibitor (or NC inh) were co-transfected in HEC-1-B cells. The IGF1R-WT (or IGF1R-MUT) and miR-34b-5p mimics (or NC miR) were co-transfected in Ishikawa cells. ***Experiment 3:*** The 3′-UTR fragments of the following genes (HOXB5, NFIX, IGF1R, MDM4, CCND1, SPSB1, SF3B3, SESN3, KIAA2013, and PRELP) were cloned into pmirGlo vectors. HEK-293 T cells were co-transfected with each type of pmirGlo vectors and miR-34b-5p mimics at 70–80% confluence. In all experiments, the luciferase activities were measured at 48-h post-transfection using the Luciferase Reporter Assay System (Promega Biotech Co., Madison, USA).

### qRT-PCR

Total RNAs were isolated from tissue samples and whole-cell lysates using Trizol (Invitrogen) and Trizol LS (Invitrogen), respectively. In EC cells, the nuclear and cytoplasmic cell fractionations were obtained using the PARIS kit (Thermo Fisher Scientific) prior to the RNA isolation. The ReverTra Ace qPCR RT Kit (Toyobo, Osaka, Japan) was used to reverse transcribe RNAs to cDNA. The reverse transcription of miRNA was performed using All-in-One™ miRNA RT-qPCR Detection Kit (GeneCopoeia Inc., Rockville, USA). The cDNAs were analyzed using 7300 Real-Time PCR System (Applied Biosystem, Foster City, USA). The primers were as follows: HOXB5 forward: 5′-AGCGCCAATTTCACCGAA-3′, HOXB5 reverse: 5′-GGCTGCTTAGCTGGCTTGC-3′; SESN3 (Hs00376220_m1, Applied Biosystems, Foster City, USA), IGF1R forward: 5′-GGCACAATTACTGCTCCAAAGAC-3′, IGF1R reverse: 5′-CAAGGCCCTTTCTCCCCAC-3′; U6 forward: 5′-CTCGCTTCGGCAGCACATATACT-3′. U6 reverse: 5′-ACGCTTCACGAATTTGCGTGTC-3′; GAPDH forward: 5′-ATCACTGCCACCCAGAAGAC-3′, GAPDH reverse: 5′-TTTCTAGACGGCAGGTCAGG-3′.

### RNA immunoprecipitation (RIP) assay

The RIP assay was performed in HEC-1-B and Ishikawa cells using the Imprint® RNA Immunoprecipitation Kit (Sigma-Aldrich) as previously described [21]. An anti-IgG antibody (Sigma-Aldrich) was used as a negative control. A mouse monoclonal anti-Argonaute2 (anti-Ago2) antibody (Sigma-Aldrich) was used as a positive control. Briefly, cells were collected, lysed in RIP lysis buffer, and incubated with anti-IgG or anti-Ago2 overnight at 4 °C. A volume of 40 μL Protein A magnetic beads were added. Total RNAs were isolated using GenElute™ Total RNA Purification Kit (Sigma-Aldrich). The enrichment of hsa_circ_0002577-WT and miR-625-5p were quantified using quantitative real-time PCR (qRT-PCR).

### Western blot

Total proteins were extracted from homogenized tissues and cell lysates using RIPA buffer containing protease inhibitors and phosphatase inhibitors (Pierce, Rockford, USA). An equal amount of protein lysates was separated on 12% SDS-PAGE and then transferred to polyvinylidene fluoride membranes. After an overnight incubation with the following primary antibodies at 4 °C: HOXB5 (1:1000, #109375, Abcam, Cambridge, UK), SESN3 (1:800, #97792, Abcam), IGF1R (0.1 μg/mL, #AF-305-SP, R&D Systems, Inc., Minneapolis, USA), protein kinase B (Akt, 1:1000, #9272, Cell Signaling), phosphorylated Akt (p-Akt, 1:1000, #9271, Cell Signaling, Danvers, USA), PCNA (PCNA, 1:1000, #18197, Abcam), p27 (1:1000, #32034, Abcam), B-cell lymphoma 2 (Bcl-2, 1:800, #59348, Abcam), E-cadherin (1:2000, #40772, Abcam), N-cadherin (1:1000, #76057, Abcam), cleaved caspase-3 (1:1000, #49822, Abcam), and GAPDH (1:2000, #37168, Abcam), the membranes were washed with Tris-Buffered Saline and stained with secondary antibody (1:2000, #6721, Abcam) for 60 min at room temperature. The images were captured using Alphalmager™ 2000 Imaging System (Alpha Innotech, USA).

### Animal study

Female BALB/c nude mice (4-week-old, Charles River Laboratories, Guangdong, China) were housed in a pathogen-free facility with a temperature of 24 ± 1 °C, a humidity of 50%, 12-h light-dark cycle, and ad libitum access to water and food. Ishikawa cells were transfected with shRNA against hsa_circ_0002577 or the control sequence. After one week of acclimation, animals were randomly assigned into two groups (*n* = 6/group): sh-circRNA and sh-NC. The sh-circRNA group was subcutaneously injected with 1 × 10^7^ sh-circRNA-transfected cells (suspended in 250 μL culture medium) into the dorsal flank. The sh-NC group was inoculated with shNC-transfected cells following the same procedure. The tumor volume was calculated every week for 6 weeks using the formula: volume = (π × length × width^2^)/6. Six weeks after inoculation, the metastasis of tumors in mice implanted with Ishikawa cells was evaluated using bioluminescence imaging as previously described [22]. Briefly, animals were anesthetized by isoflurane and then administered with D-luciferin potassium salt (Biosynth, Staad, Switzerland) at the dosage of 150 mg/kg via intraperitoneal injection. Fifteen minutes later, mouse was placed in a supine position. Bioluminescence imaging was performed using an in vivo imaging system (IVIS, PerkinElmer, Waltham, USA). The intensity of signal was reported as radiance (photons/s/cm^2^/steradian). Then all animals were euthanized and tumor xenografts were harvested and weighted. The tumor samples were sectioned and stained for IGF1R (#110025, Abcam), Ki-67 (#15580, Abcam), E-cadherin, and CD31 (#28364, Abcam) using immunohistochemistry method (magnification 40×). The sectioned slides were stained with TUNEL reagent for apoptosis. Hematoxylin and eosin (H&E) staining was also performed to detect metastatic pulmonary nodules and the density of microvessels. All procedures were approved by the Medical Ethics Committee of the Shengjing Hospital Affiliated to China Medical University, and performed according to the Guide for the Care and Use of Laboratory Animals [23].

### Statistical analysis

Data are shown as mean ± standard deviation and analyzed using software SPSS (version 24.0). All cell culture experiments in this study were performed in triplicate and repeated three times. One-way analysis of variance (ANOVA) was used to evaluate statistical significance. Survival data was analyzed using the Kaplan-Meier method. The linear correlation coefficient was used to estimate the correlation in the expression levels of hsa_circ_0002577 vs. miR-625-5p, hsa_circ_0002577 vs. IGF1R, and miR-625-5p vs. IGF1R. A value of *p* < 0.05 was considered statistically significant. * *p* < 0.05, ** *p* < 0.01.

## Results

### Hsa_circ_0002577 expression was increased in EC tissues and cells lines

**Fig. 1:**
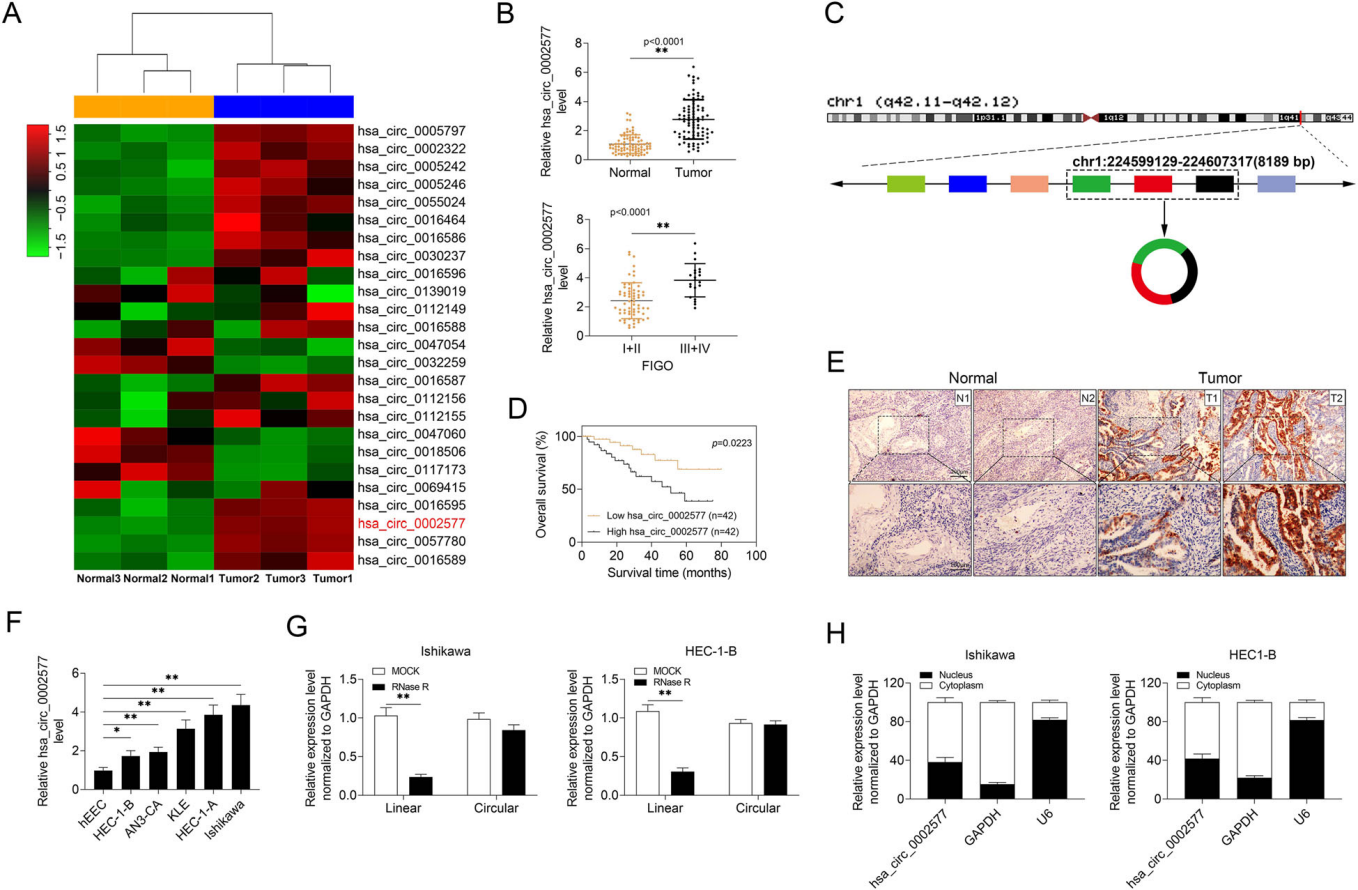
The expression level of hsa_circ_0002577 in EC tissues and cell lines and its clinical significance. **a** The relative expression levels of circRNAs in EC and paired adjacent normal tissue samples (*n* = 3) were analyzed using qRT-PCR and the expression heat map was generated using program R. **b** The expression of hsa_circ_0002577 in EC vs. normal tissues (*n* = 84) and in patients with FIGO stage I/II vs. stage III/IV. **c** Schematic illustration of hsa_circ_0002577 formation via the circularization of a segment in WDR26 gene (Genome Informatics, https://genome.ucsc.edu). **d** The Kaplan-Meier survival analysis of EC patients with high (*n* = 42) and low (*n* = 42) expressions of low hsa_circ_0002577. **e** Representative images of FISH analysis of hsa_circ_0002577 in normal and EC tissues. **f** The relative expression of hsa_circ_0002577 in human endometrial endothelial cell (hEEC) and EC cell lines (HEC-1-B, AN3-CA, KLE, HEC1-A, and Ishikawa). **g** The circular RNAs in Ishikawa and HEC-1-B cells were enriched by RNase R treatment. The expressions of linear and circular RNAs in cells received RNase R or mock treatment were analyzed. **h** The relative expression of hsa_circ_0002577 in the nucleus and cytoplasm of EC cells were analyzed. GAPDH was used as cytoplasmic control and U6 was used as nuclear control

To identify differentially expressed circRNAs in EC, an expression heat map was generated using three pairs of EC and adjacent normal tissues. The results showed that hsa_circ_0002577, hsa_circ_0005797, hsa_circ_0057780, and hsa_circ_0016595 were the top upregulated circRNAs between EC and noncancerous tissues (Fig. 1 a). Then we measured the expressions of these circRNAs in 84 paired EC and normal tissue samples, and found that hsa_circ_0002577 was the most upregulated one in EC tissues (Fig. 1 b, Supplementary Figure 1A). Also, compared to the samples collected from patients at stage I/II, the expression of hsa_circ_0002577 was significantly higher in advanced EC tissues (III/IV) (Fig. 1 b). Therefore, we focused on hsa_circ_0002577, which was generated from WDR26 gene. (Fig. 1 c). Further analysis showed that the low expression of hsa_circ_0002577 was correlated with significantly better overall survival compared with high expression (Fig. 1 d). By analyzing the clinicopathological significance of hsa_circ_0002577 in patients, we also found that the expression of hsa_circ_0002577 in EC tissues was positively and significantly associated with high histological grade of tumor, lymph node metastasis (LNM) staging, and lymph vascular space invasion (LVS) (Table 1). The FISH analysis showed that hsa_circ_0002577 was prominently overexpressed in EC tissues in comparison to paired normal samples (Fig. 1 e). Next, we measured the expression of the target circRNA in EC cell lines. Compared to hEEC, all tested EC cells exhibited a significantly higher level of hsa_circ_0002577, among which HEC-1-B cells had the lowest expression and Ishikawa cells showed the highest hsa_circ_0002577 level (Fig. 1 f). Then RNase R treatment was performed to enrich circRNAs in Ishikawa and HEC-1-B cells followed by the examination of RNA (linear and circular) expressions. The level of linear RNAs in RNase R-treated group was markedly lower than that in the mock group, whereas the expression of circRNAs was not affected by RNase R treatment (Fig. 1 g). Via the detection of hsa_circ_0002577 level in the nuclear and cytoplasmic fractionations of EC cells, we found that hsa_circ_0002577 was abundantly expressed in cytoplasm (Fig. 1 h). The above findings indicated that the upregulation of hsa_circ_0002577 may exert an oncogenic effect on the progression of EC.

**Table 1:** Relationship between hsa_circ_0002577 and clinico-pathological parameters

Parameters	Number of patients	hsa_circ_0002577 expression		Positive rate (%)	*P* value
		Low (< median)	High (≥ median)		
Number	84	42	42		
Age (years)					
≥ Mean (55)	58	28	30	69	0.637
< Mean (55)	26	14	12	31	
FIGO stage					
I-II	63	37	26	75	0.006**
III-IV	21	5	16	25	
Pathological type					
Endometrioid	68	36	32	81	0.266
Non-endometrioid	16	6	10	19	
Histological grade					
Grade 1	45	30	15	54	0.002**
Grade 2	27	10	17	32	
Grade 3	12	2	10	14	
LNM					
Positive	22	5	17	26	0.003**
Negative	62	37	25	74	
LVS					
Positive	26	6	20	31	0.001**
Negative	58	36	22	69	
Depth of myometrial invasion					
≤ 1/2	69	37	32	82	0.154
> 1/2	15	5	10	18	
ER expression					
Positive	19	7	12	23	0.192
Negative	65	35	30	77	
FR expression					
Positive	21	7	14	25	0.078
Negative	63	35	28	75	

### Hsa_circ_0002577 induced EC cell proliferation

**Fig. 2:**
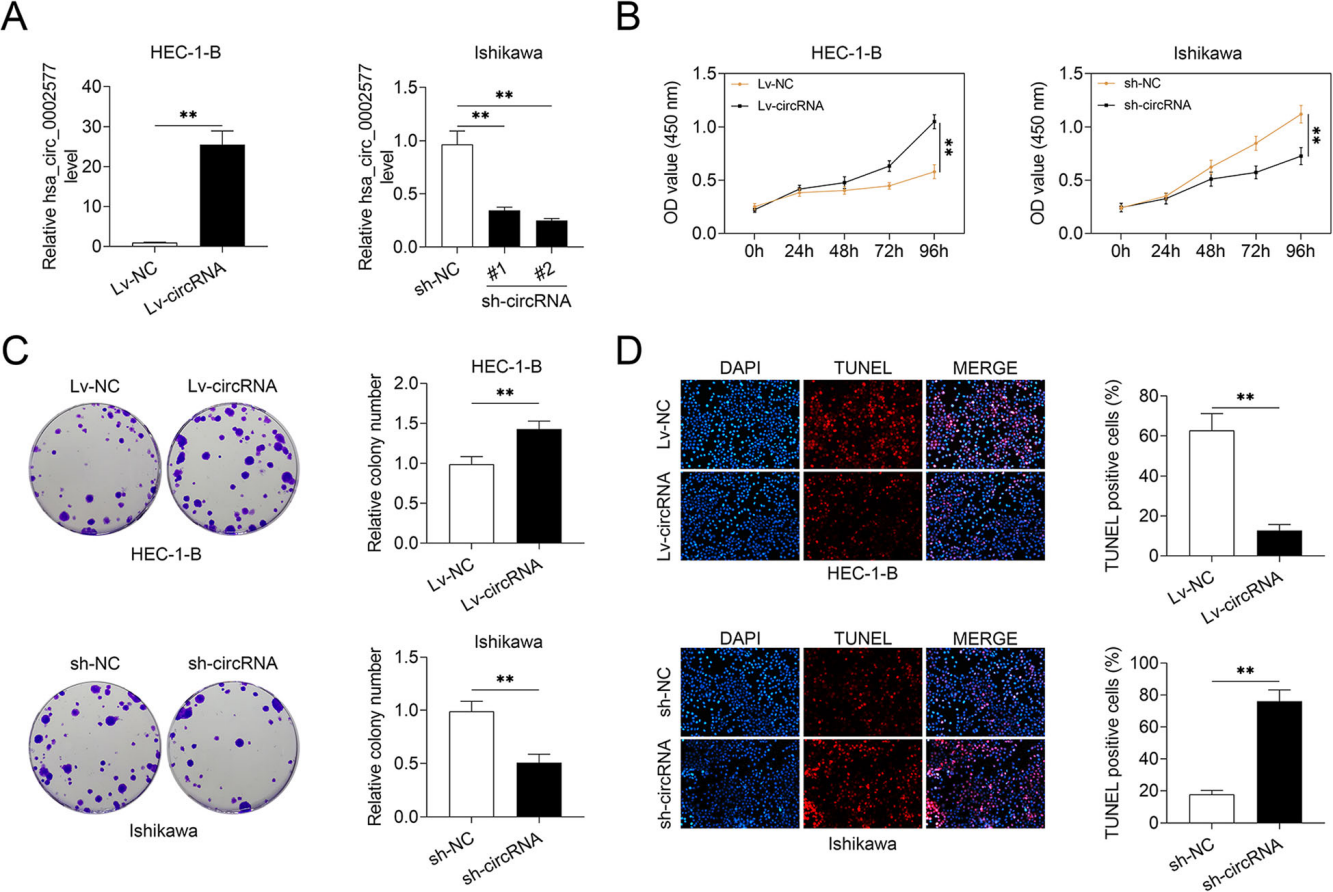
Effect of hsa_circ_0002577 on EC cell proliferation. HEC-1-B cells were transfected with recombinant lentiviral vectors expressing hsa_circ_0002577 (Lv-circRNA) or the control vector (Lv-NC). Ishikawa cells were transfected with two shRNAs against hsa_circ_0002577 (sh-circRNA#1 and #2) or the negative control sequences. **a** The relative expression of hsa_circ_0002577 in transfected EC cells was detected using qRT-PCR. **b** CCK-8 assay was performed to evaluate cell proliferation in transfected EC cells at indicated time points (0, 24, 48, 72, and 96 h) post-transfection. The OD value at 450 nm was shown. **c** The number of colonies in transfected HEC-1-B and Ishikawa cells was measured 10 days after transfection. **d** TUNEL and DAPI staining was performed to detect apoptotic cell death in EC cells. The percentage of TUNEL-positive cells was calculated

To explore the potential regulatory role of hsa_circ_0002577 in EC cells, we transfected HEC-1-B cells with lentiviral vectors expressing hsa_circ_0002577 (or the control vector), and transfected Ishikawa cells with two shRNAs against hsa_circ_0002577 (or the control sequences). The delivery of Lv-circRNA significantly induced the overexpression of hsa_circ_0002577 in HEC-1-B cells, while sh-circRNA#1 and #2 effectively decreased the level of hsa_circ_0002577 in Ishikawa cells (Fig. 2 a). As sh-circRNA#2 showed more potent knockdown efficiency than sh-circRNA#1, sh-circRNA#2 was used in further analyses, named sh-circRNA. The CCK-8 proliferation assay showed that the upregulation of hsa_circ_0002577 strongly induced the proliferation capacity of HEC-1-B cells at 96 h post-transfection, whereas the knockdown of target circRNA efficiently suppressed the proliferation of Ishikawa cells (Fig. 2 b). Consistently, the number of colonies was significantly increased in Lv-circRNA-transfected cells compared to the controls, but reduced in cells with hsa_circ_0002577 deficiency (Fig. 2 c). Compared to control vector-transfected cells, the number of TUNEL-positive cells was significantly decreased in HEC-1-B cells transfected with Lv-circRNA, indicating that hsa_circ_0002577 overexpression rescued EC cells from apoptosis. Meanwhile, extensive apoptosis was observed in EC cells with insufficient hsa_circ_0002577 expression (Fig. 2 d). These findings suggested that EC cell proliferation and apoptosis were mediated by the level of hsa_circ_0002577.

### Hsa_circ_0002577 promoted EC cell migration/invasion

**Fig. 3:**
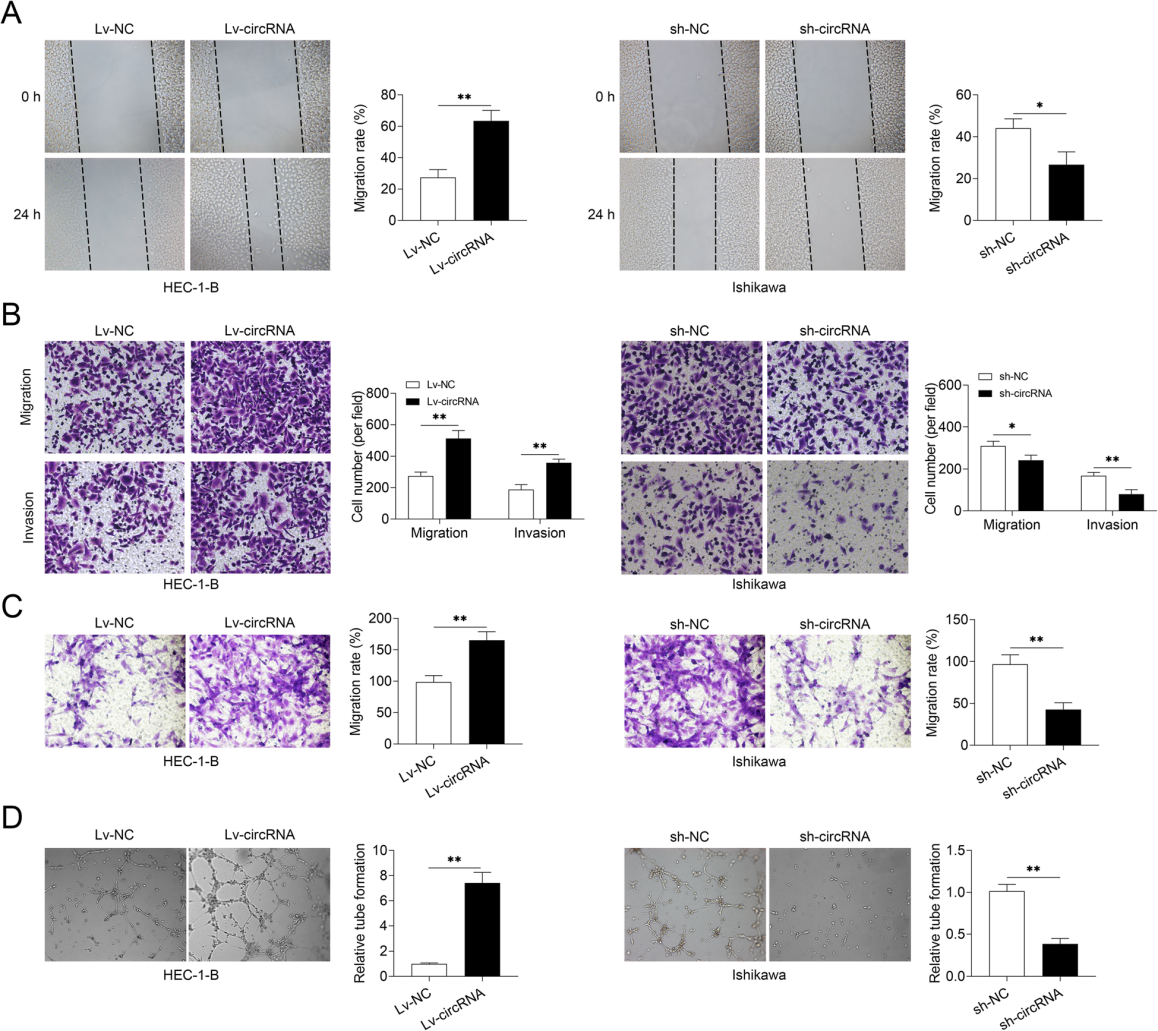
Effect of hsa_circ_0002577 on EC cell migration/invasion. HEC-1-B and Ishikawa cells were transfected with designated vectors or sequences. **a** The migration capability of cells after transfection was evaluated by wound healing assay. The relative migration rate was analyzed. **b** Transwell assay was performed at 48 h post-transfection and the number of migrated and invaded cells were counted. **c** Cell supernatants were collected from transfected HEC-1-B and Ishikawa cells. HUVECs were cultured with a mixture of EC cell supernatants and ECGM for 4 days. Then Transwell assay was performed and the migration rate was calculated. **d** Cell supernatants were collected from transfected HEC-1-B and Ishikawa cells. HUVECs were cultured with a mixture of EC cell supernatants and ECGM for 4 days. HUVECs treated with different cell supernatants were transferred to Matrigel-coated wells and incubated at 37 °C for 24 h. The number of branches in each group of cells was quantified

Furthermore, cells overexpressing hsa_circ_0002577 significantly accelerated wound closure as compared to Lv-NC-transfected group in wound healing assay, whereas the knockdown of hsa_circ_0002577 repressed cell migration, as shown by significantly decreased migration rate compared with the controls (Fig. 3 a). Also, a higher number of migrated and invaded cells were found in Lv-circRNA group as compared to the control cells in the Transwell assay. The hsa_circ_0002577 deficiency, however, impeded the migration/invasion of EC cells (Fig. 3 b). Angiogenesis is a key event in tumor development, in which new capillary networks are formed by vascular endothelial cells, facilitating the delivery of oxygen and nutrients to the tumor [24]. To explore whether hsa_circ_0002577 might affect the formation of tubule-like endothelial structures, we cultured HUVECs with the cell supernatants collected from transfected EC cells. The supernatants of cells overexpressing hsa_circ_0002577 significantly induced HUVECs migration (Fig. 3 c) and increased the number of branches in HUVECs (Fig. 3 d) as compared to the control cells. On the contrary, the supernatants of cells with hsa_circ_0002577 deficiency suppressed the migration and tubule formation of HUVECs. These data implied that hsa_circ_0002577 not only induced the migration/invasion of EC cells, but also promoted the tubule formation of HUVECs.

### Hsa_circ_0002577 is a miR-625-5p sponge

**Fig. 4:**
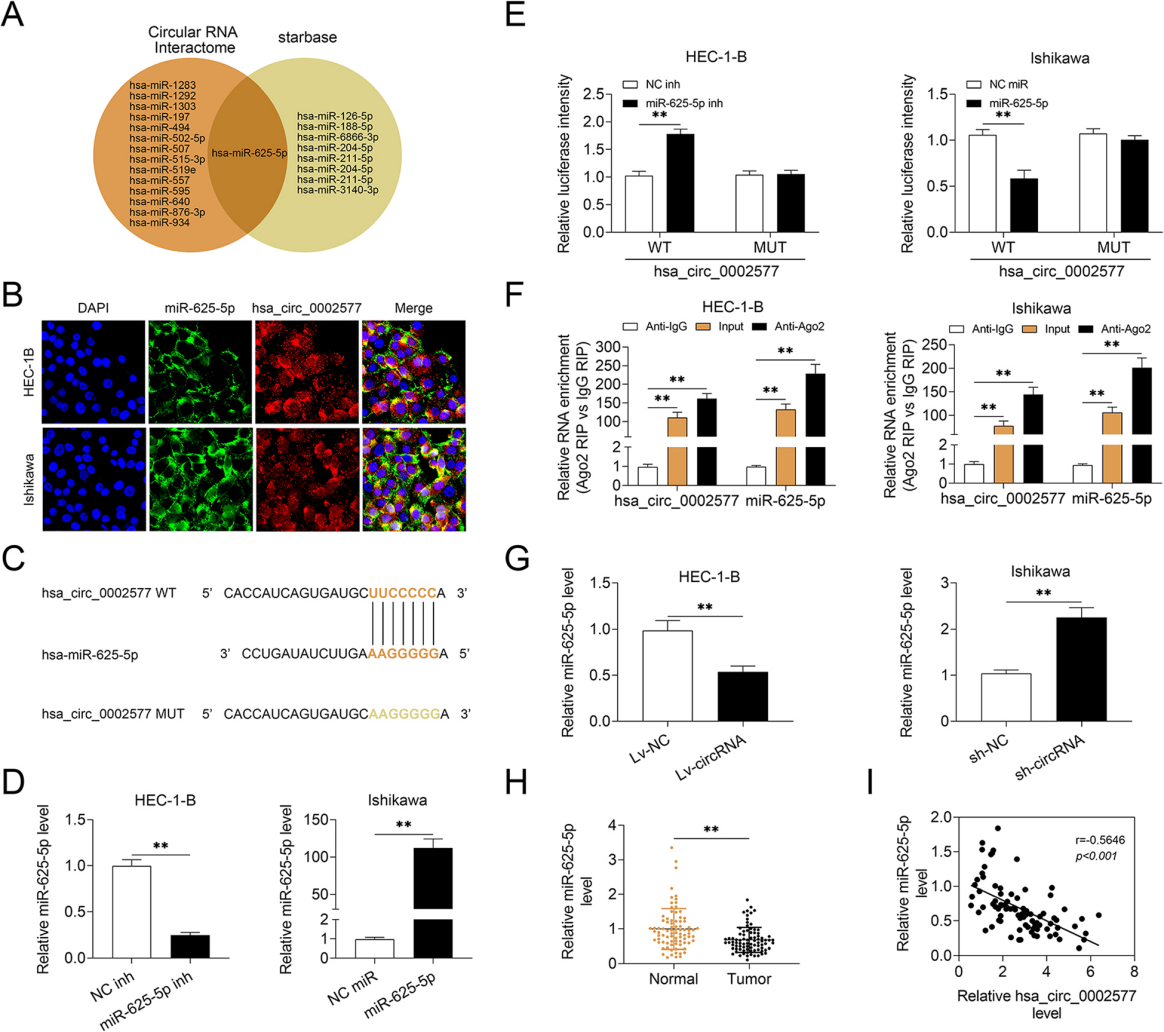
Hsa_circ_0002577 acts as a sponge for miR-625-5p. **a** The biding sites of hsa_circ_0002577 for miRNAs were predicted using Circular RNA Interactome (https://circinteractome.nia.nih.gov/) and Starbase (http://starbase.sysu.edu.cn/index.php). Hsa_circ_0002577 was shown to have a putative binding site for miR-625-5p in both databases. **b** Representative fluorescence images of HEC-1-B and Ishikawa cells stained with DAPI, miR-625-5p probe, and hsa_circ_0002577 probe. **c** The putative binding site of hsa_circ_0002577 for miR-625-5p (hsa_circ_0002577-WT) and the designed mutant sequence (hsa_circ_0002577-MUT) were shown. **d** HEC-1-B cells were transfected with miR-625-5p inhibitor or control inhibitor (NC inh). Ishikawa cells were transfected with miR-625-5p mimics or control mimics (NC miR). The relative expression level of miR-625-5p was measured using qRT-PCR. **e** HEC-1-B cells were co-transfected with miR-625-5p inhibitor (or NC inh) and hsa_circ_0002577-WT (or hsa_circ_0002577-MUT). Ishikawa cells were co-transfected with miR-625-5p mimics (or NC miR) and hsa_circ_0002577-WT (or hsa_circ_0002577-MUT). Dual-luciferase reporter assay was performed at 48 h post-transfection to assess the luciferase activity. **f** The RIP assay was performed in EC cells with an anti-Ago2 antibody as a positive control and an anti-IgG antibody as a negative control. The relative expressions of hsa_circ_0002577 and miR-625-5p were detected using qRT-PCR. **g** The relative expression level of miR-625-5p in HEC-1-B cells transfected Lv-circRNA (or Lv-NC) and Ishikawa cells transfected with sh-circRNA (or sh-NC) was quantified by qRT-PCR. **h** The expression of miR-625-5p in EC and normal tissue samples (*n* = 84). **i** The correlation between hsa_circ_0002577 level and miR-625-5p expression was estimated by linear correlation coefficient

To investigate the underlying mechanisms involved in the regulation of EC tumorigenesis by hsa_circ_0002577, we searched for the potential bindings sites of hsa_circ_0002577 for miRNAs. The bioinformatic prediction analysis from two databases showed that hsa_circ_0002577 had a putative binding site for miR-625-5p (Fig. 4 a). The fluorescence staining of EC cells with DAPI, miR-625-5p probe, and hsa_circ_0002577 probe demonstrated that both miR-625-5p and hsa_circ_0002577 were predominantly expressed in cytoplasm (Fig. 4 b). We speculated that hsa_circ_0002577 might competitively binds to miR-625-5p as a miRNA sponge to regulate its expression and function in cells. The putative binding site of hsa_circ_0002577 for miR-625-5p and a mutant sequence were shown (Fig. 4 c). The transfection with miR-625-5p inhibitor significantly downregulated the expression of miR-625-5p in HEC-1-B cells, while Ishikawa cells introduced with miR-625-5p mimics demonstrated a remarkably elevated level of miR-625-5p compared to the group transfected with control mimics (Fig. 4 d). The luciferase activity of hsa_circ_0002577-WT was markedly upregulated in EC cells delivered with miR-625-5p inhibitor but decreased in miR-34b-5p mimics-transfected group, as compared to their corresponding controls. Cells transfected with the mutant sequence exhibited no difference in the luciferase activity between the mimics/inhibitor-transfected cells and the control groups (Fig. 4 e). The RIP assay using the Ago2-sepecific antibody showed that both miR-625-5p and hsa_circ_0002577 were enriched in Ago2 immunoprecipitation, confirming their interaction in EC cells (Fig. 4 f). Moreover, the expression of miR-625-5p in HEC-1-B cells transfected with Lv-circRNA was significantly higher than that in control cells. Hsa_circ_0002577 knockdown, however, promoted the expression of miR-625-5p in EC cells (Fig. 4 g). A significant variation in the level of miR-625-5p was also observed in patients’ samples. Tumor tissues showed significantly lower expression of miR-625-5p in comparison to paired normal samples (Fig. 4 h). The hsa_circ_0002577 level was inversely and significantly correlated with miR-625-5p expression (Fig. 4 i). Additionally, EC patients with low expression of miR-625-5p showed significantly better overall survival compared with those with high miR-625-5p expression (Supplementary Figure 1B). The above results indicated that hsa_circ_0002577 reversely regulated the expression of miR-625-5p in EC cells and tissues.

### Hsa_circ_0002577 induced the upregulation of IGF1R via targeting miR-625-5p

**Fig. 5:**
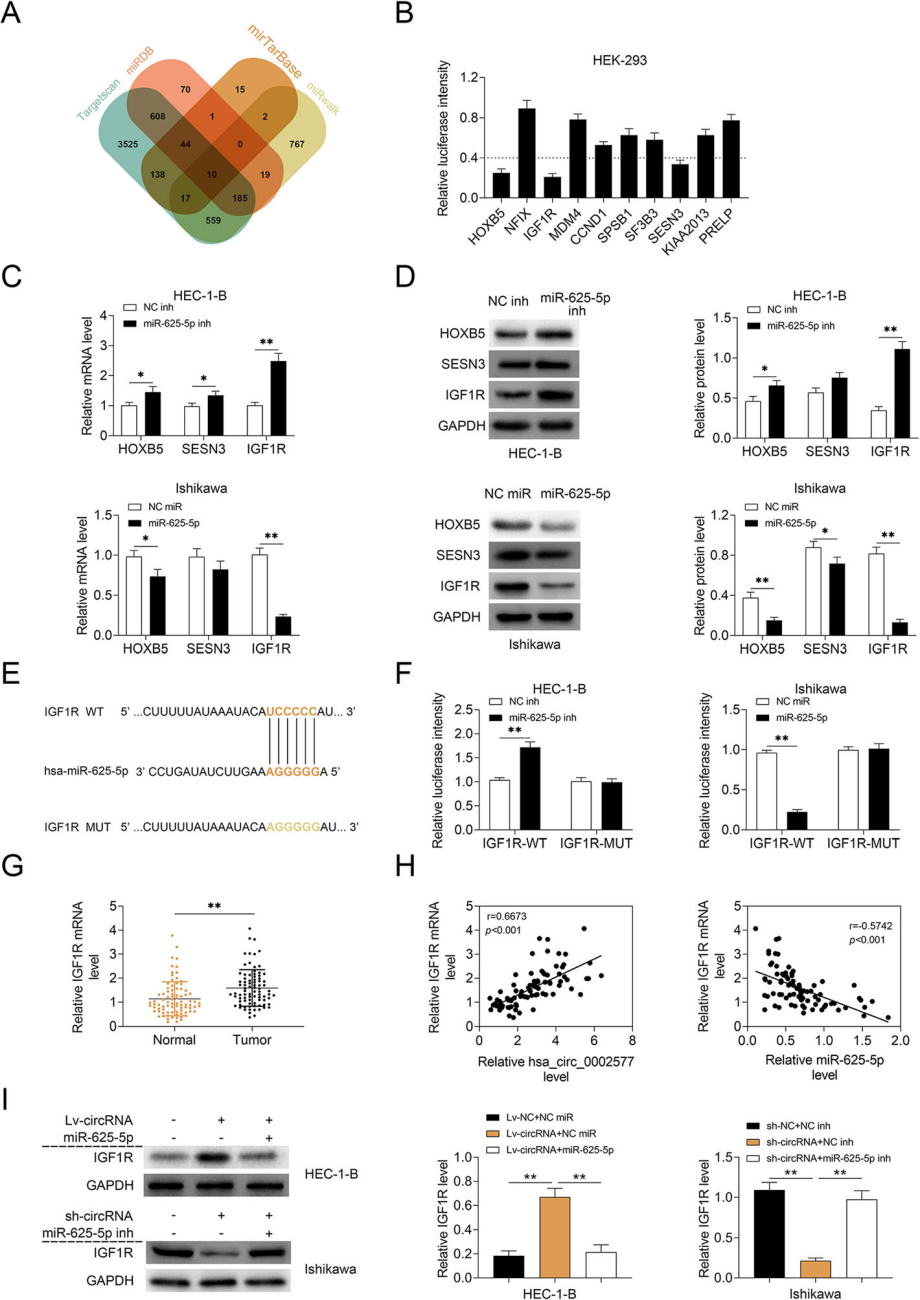
Hsa_circ_0002577 regulates the expression of IGF1R via targeting miR-625-5p. **a** The target genes of miR-625-5p was predicted using Targetscan (www.targetscan.org), mirTarBase (http://mirtarbase.mbc.nctu.edu.tw/php/index.php), miRDB (www.mirdb.org), and miRwalk (http://zmf.umm.uni-heidelberg.de/apps/zmf/mirwalk/index.html). Ten genes were shown as potential target genes of miR-625-5p. **b** The luciferase vectors containing the 3′-UTR fragments of these genes (HOXB5, NFIX, IGF1R, MDM4, CCND1, SPSB1, SF3B3, SESN3, KIAA2013, and PRELP) were transfected into HEK-293 T cells. The luciferase activities were measured at 48 h post-transfection. **c** HEC-1-B cells were transfected with miR-625-5p inhibitor or control inhibitor (NC inh). Ishikawa cells were transfected with miR-625-5p mimics or control mimics (NC miR). The relative mRNA and expressions of HOXB5, SESN3, and IGF1R were analyzed using qRT-PCR. **d** The protein levels of these genes were measured using Western blot. **e** The putative binding site of IGF1R for miR-625-5p (IGF1R-WT) and the mutant sequence (IGF1R-MUT) were shown. **f** HEC-1-B cells were co-transfected with miR-625-5p inhibitor (or NC inh) and IGF1R-WT (or IGF1R-MUT). Ishikawa cells were co-transfected with miR-625-5p mimics (or NC miR) and IGF1R-WT (or IGF1R-MUT). The luciferase activity was determined 48 h after transfection by dual-luciferase reporter assay. **g** The mRNA expression of IGF1R in EC and normal tissues (*n* = 84). **h** The linear correlation coefficient was performed to estimate the correlation in the expression levels of hsa_circ_0002577 vs. IGF1R and miR-625-5p vs. miR-625-5p IGF1R. **i** HEC-1-B cells were co-transfected with Lv-circRNA (or Lv-NC) and miR-625-5p mimics (or NC miR). Ishikawa cells were co-transfected with sh-circRNA (or sh-NC) and miR-625-5p mimics (or NC miR). The protein expression level of IGF1R was measured by Western blot at 48 h post-transfection

To further explore the involvement of miR-625-5p in hsa_circ_0002577-mediated EC pathogenesis, we predicted the downstream targets of miR-625-5p in four bioinformatic databases. Results showed that ten genes might be the potential targets of miR-625-5p (Fig. 5 a). We transfected HEK-293 T cells with luciferase vectors containing the 3′-UTR fragments of these genes and miR-34b-5p mimics. Cells transfected with vectors containing *HOXB5*, *IGF1R*, and *SESN3* genes showed luciferase activity lower than 40% (Fig. 5 b). In HEC-1-B cells, the transfection with miR-625-5p inhibitor significantly increased the transcription of these genes compared to the controls, with the most robust induction in *IGF1R*. In Ishikawa cells, the overexpression of miR-625-5p significantly suppressed the mRNA expressions of *HOXB5* and *IGF1R*, but not *SESN3*, with the greatest inhibition in *IGF1R* (Fig. 5 c). Consistently, Western blot analysis of both miR-625-5p-overexpressed and -deficient cells showed that the biggest alteration in the protein expression of these genes was seen in IGF1R (Fig. 5 d). The putative binding site of IGF1R for miR-625-5p and a mutant sequence were shown (Fig. 5 e). The luciferase intensity of IGF1R-WT was increased in cells transfected with miR-625-5p inhibitor but decreased in the ones delivered with miR-34b-5p mimics, as compared to their corresponding controls (Fig. 5 f). The mRNA expression of *IGF1R* in tumor tissues was significantly higher than that in normal tissues (Fig. 5 g). Also, the mRNA expression of *IGF1R* in was positively correlated with hsa_circ_0002577 level and inversely correlated with miR-185-5p expression with statistical significance (Fig. 5 h). Additionally, hsa_circ_0002577 overexpression in HEC-1-B cells significantly increased the protein level of IGF1R, whereas the co-transfection with both Lv-circRNA and miR-625-5p mimics eliminated the effect of hsa_circ_0002577 on IGF1R. In Ishikawa cells, the co-transfection with sh-circRNA and miR-625-5p mimics reversed the inhibitory effect of hsa_circ_0002577 deficiency on IGF1R expression (Fig. 5 i). These data suggested that hsa_circ_0002577 induced the expression of IGF1R via inhibiting miR-625-5p.

### Hsa_circ_0002577 induced EC cell proliferation and migration by regulating IGF1R and PI3K/Akt pathway

**Fig. 6:**
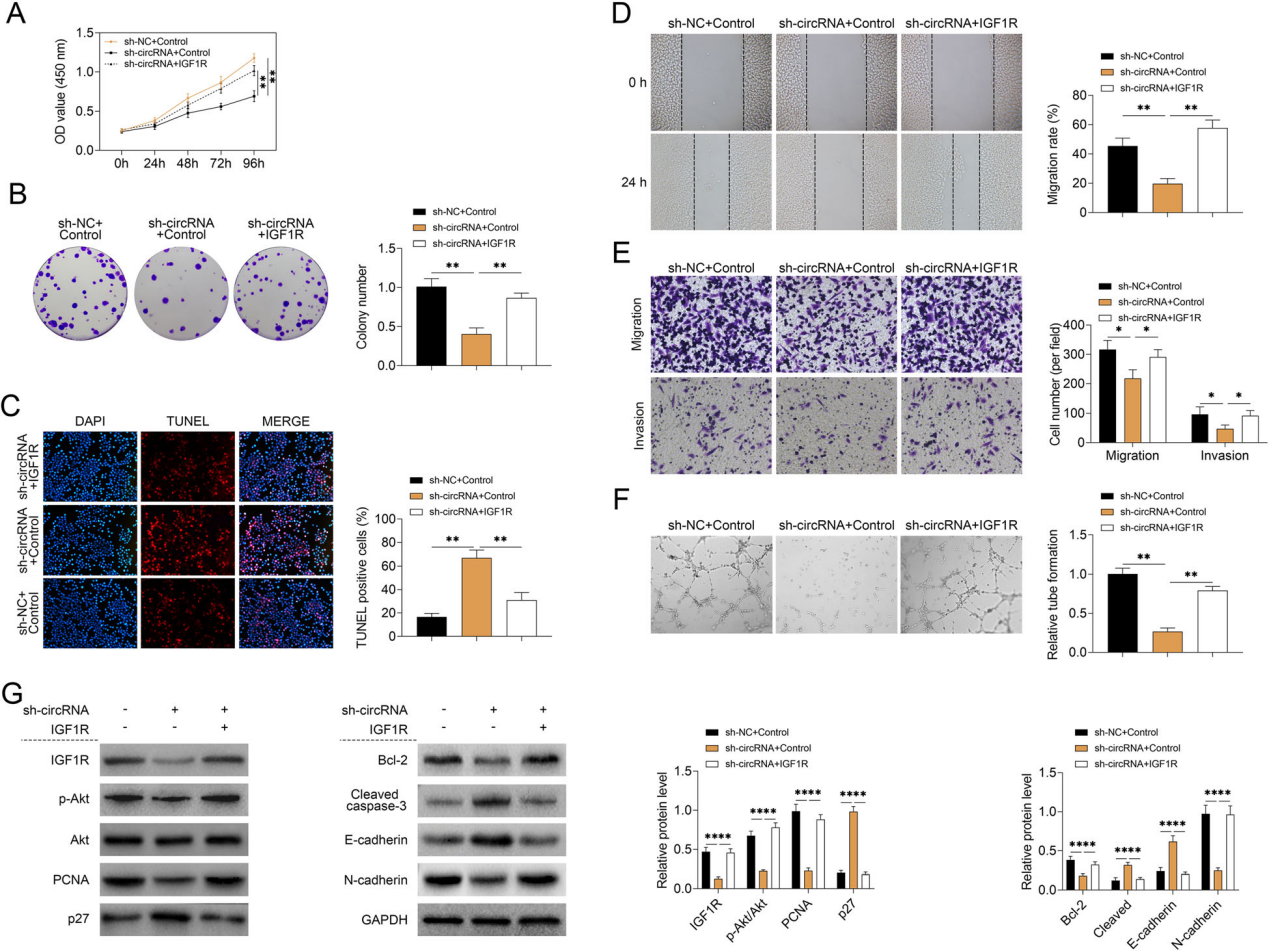
Hsa_circ_0002577 regulates EC cell proliferation and migration via the modulation of IGF1R and PI3K/Akt signaling pathway. Ishikawa cells were co-transfected with sh-circRNA (or sh-NC) and lentiviral vectors expressing IGF1R (or control vectors). **a** Cell proliferation at indicated time points (0, 24, 48, 72, and 96 h) post-transfection was evaluated using CCK-8 assay. **b** The number of colonies was measured 10 days after transfection. **c** The apoptosis of transfected Ishikawa cells was detected using TUNEL and DAPI staining. The percentage of TUNEL-positive cells was calculated. The migration capability of cells after transfection was assessed by **d** wound healing assay and **e** Transwell assay. **f** HUVECs were cultured with ECGM containing the supernatants collected from Ishikawa cells co-transfected with sh-circRNA (or sh-NC) and lentiviral vectors expressing IGF1R (or control vectors) for 4 days. Then HUVECs were transferred to Matrigel-coated wells and incubated at 37 °C for 24 h. The number of branches in each group of cells was quantified. **g** The protein expressions of IGF1R, p-Akt, Akt, PCNA, p27, Bcl-2, cleaved caspase-3, E-cadherin, and N-cadherin were measured by Western blot and normalized to GAPDH

To ascertain that hsa_circ_0002577 regulated EC progression by targeting IGF1R, we co-transfected Ishikawa cells with sh-circRNA (or sh-NC) and lentiviral vectors expressing IGF1R (or control vectors). As shown above, hsa_circ_0002577 knockdown significantly suppressed cell proliferation and induced apoptosis in Ishikawa cells. By introducing cells with IGF1R-overexpressing vectors, the proliferation capacity (Fig. 6 a) and the number of colonies (Fig. 6 b) was significantly increased. Also, the upregulation of IGF1R in hsa_circ_0002577 deficient cells significantly reduced the quantity of apoptotic cells (Fig. 6 c). In the analyses of cell migration/invasion, the group delivered with both IGF1R-overexpression vector and sh-circRNA showed significantly lower migration rate (Fig. 6 d) and decreased number of migrated and invaded cells (Fig. 6 e) as compared to the cells with insufficient hsa_circ_0002577 expression. To examine the effect of IGF1R on the formation of tubule-like endothelial structure, HUVECs were cultured with ECGM containing the supernatants collected from Ishikawa cells co-transfected with sh-circRNA (or sh-NC) and lentiviral vectors expressing IGF1R. The overexpression of IGF1R significantly promoted tube formation in cells with insufficient hsa_circ_0002577 expression (Fig. 6 f). Furthermore, the expressions of proteins related to the PI3K/Akt signaling pathway were analyzed. The expressions of IGF1R, p-Akt/Akt, PCNA, Bcl-2, and N-cadherin were significantly reduced by the knockdown of hsa_circ_0002577, but restored to the normal level by excessive production of IGF1R. The deficiency of hsa_circ_0002577 elevated the levels of p27, cleaved caspase-3, and E-cadherin in Ishikawa cells, which were then inhibited by IGF1R overexpression (Fig. 6 g). The above findings indicated that hsa_circ_0002577 induced EC cell proliferation and migration by regulating IGF1R and PI3K/Akt signaling pathway.

### Knockdown of hsa_circ_0002577 suppressed tumor growth and migration in vivo

**Fig. 7:**
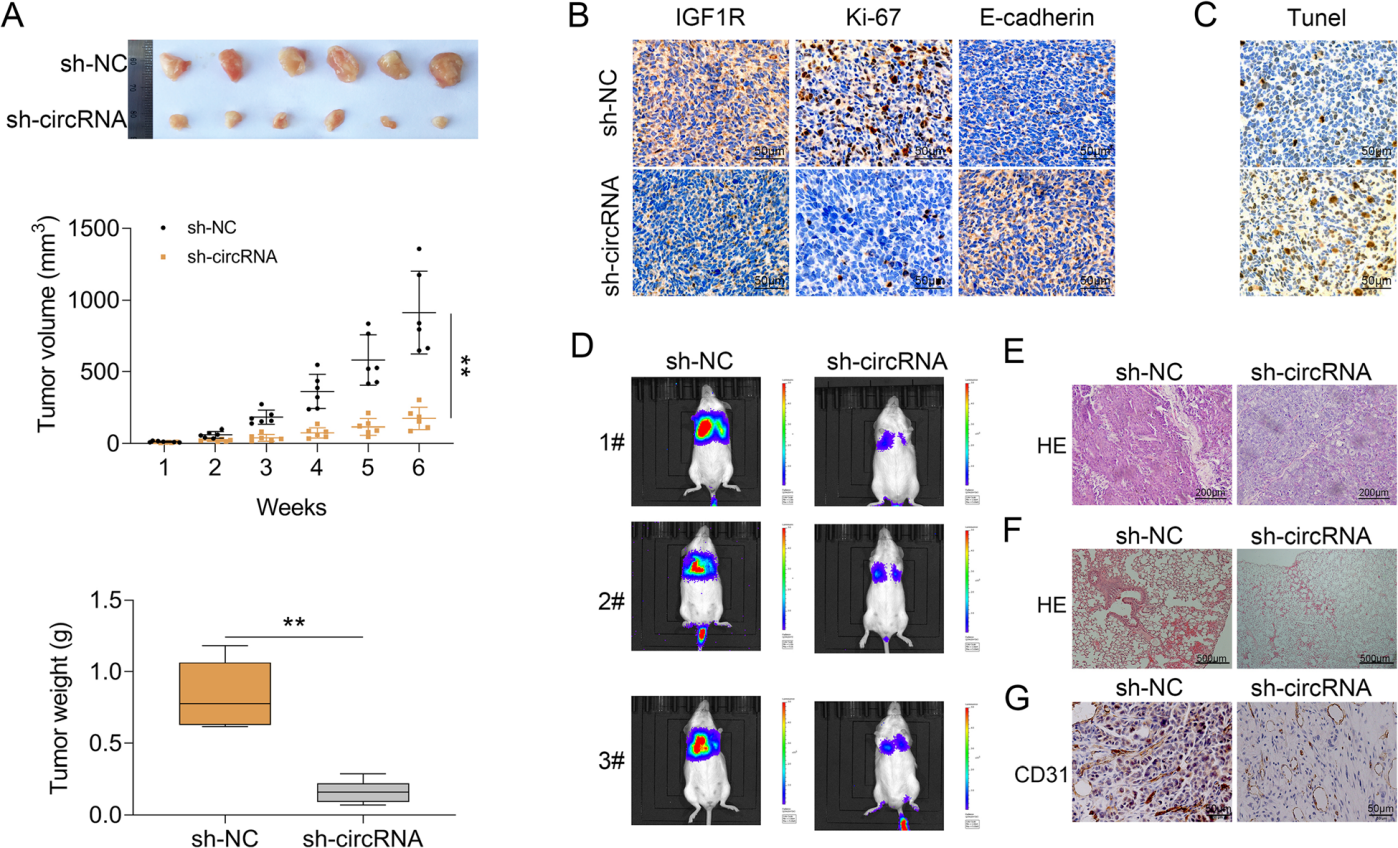
Effect of hsa_circ_0002577 knockdown on tumor growth and metastasis in EC mouse model. Four-week-old female BALB/c nude mice were subcutaneously injected with sh-circRNA or sh-NC-transfected cells into the dorsal flank. **a** Six weeks after inoculation, tumors were removed from all mice (*n* = 6 per group). The tumor volume was measured every week for 6 weeks. The average tumor weight of both groups was shown. **b** Tumor tissue samples were sectioned and stained for IGF1R, Ki-67, E-cadherin by immunohistochemistry. **c** TUNEL staining was performed to detect apoptosis. **d** The metastasis of tumor in mice implanted with Ishikawa cells was evaluated using bioluminescence imaging. The intensity of luminescence signal was shown as radiance (photons/s/cm^2^/steradian) with a color bar. **e** The metastatic pulmonary nodules and **f** the density of microvessels were visualized using H&E staining. **g** Tumor tissue sections were analyzed for CD31 using immunohistochemistry

Finally, we evaluated the regulatory effects of hsa_circ_0002577 in vivo using a mouse EC xenograft model. Ishikawa cells were transfected with sh-circRNA or control shRNA (sh-NC) and then inoculated into nude mice subcutaneously. Compared to the control group, mice injected with hsa_circ_0002577-deficient cells displayed significantly decreased tumor volume and weight (Fig. 7 a). The expressions of IGF1R and Ki-67 (a cell proliferation marker) in tumor samples collected from sh-circRNA group were reduced compared to the control mice. The expression of E-cadherin (a differentiation and invasiveness marker), however, was increased by the knockdown of hsa_circ_0002577 (Fig. 7 b). The sh-circRNA group also showed more apoptotic cells (Fig. 7 c). The bioluminescence imaging showed that hsa_circ_0002577 deficiency ameliorated the growth and metastasis of tumor in mice implanted with Ishikawa cells (Fig. 7 d). The insufficient expression of hsa_circ_0002577 also decreased the number of metastatic pulmonary nodules (Fig. 7 e) and the density of microvessels (Fig. 7 f) in mice. In addition, compared to the control mice, the sh-circRNA group showed less stained CD31-positive cells in tumor tissue sections (Fig. 7 g). Taken together, these results demonstrated that hsa_circ_0002577 knockdown delayed the tumor growth and metastasis in the EC mouse model.

## Discussion

**Fig. 8:**
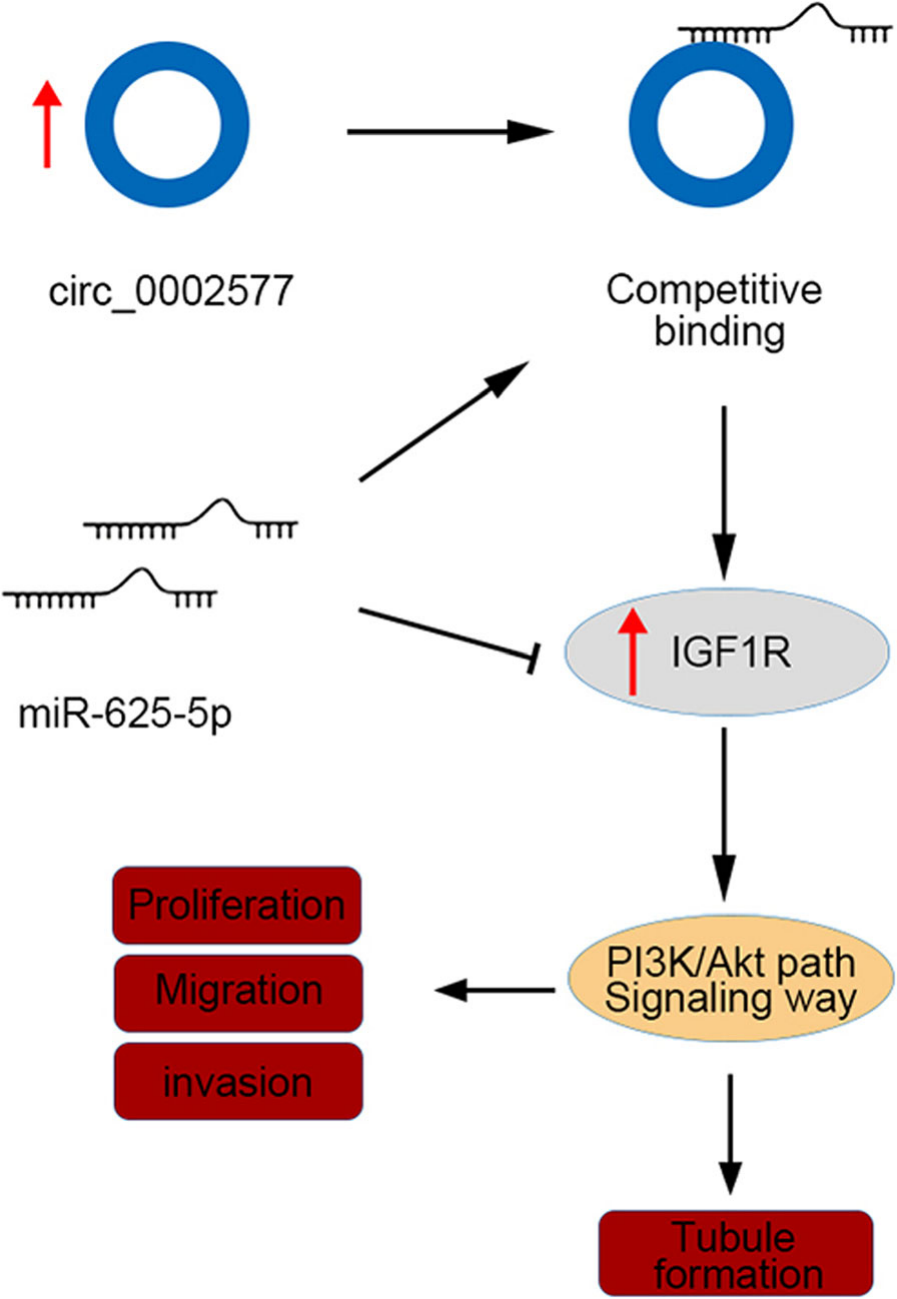
The hypothetical mechanism for the regulatory effects of circRNA hsa_circ_002577 in EC. Hsa_circ_002577 competitively binds to miR-625-5p, resulting in the upregulation of IGF1R and subsequent activation of PI3K/Akt signaling pathway, which eventually leads to the proliferation, migration, and invasion of EC cells, and the tubule formation in HUVECs

Targeted therapies that affect tumor cell growth, apoptosis, signal transduction, receptor activation, and epigenetic modifications are considered a breakthrough in human cancer treatment [25]. Noncoding RNAs are gaining significant importance in this area due to their clinical and functional relevance in cancer progression [26]. Despite the high survival rate in EC patients at early-stage, the prognosis of women with advanced or recurrent EC remains poor and the treatment options for them are limited [27]. Our study reported an upregulation of hsa_circ_0002577 in EC tissue samples and cell lines as compared to normal controls. Hsa_circ_0002577 adversely affected EC progression in vitro and in a mouse xenograft model via competitively binding to miR-625-5p, inducing the expression of IGF1R and subsequently activating PI3K/Akt signaling pathway (Fig. 8).

CircRNAs are highly conserved and stable noncoding RNAs that attracted considerable attention recently attributed to their potential functional capacity in cancer development [28]. They carry multiple miRNA binding sites and thus mediate the activity of target miRNA by competitively binding it, thereby suppressing the downstream transcriptions [29]. Circ-ABCB10 promoted the tumorigenesis of breast cancer via sponging miR-1271 [30]. The migration/invasion of bladder cancer cells and the production of heparanase were inhibited by circHIPK3 through sponging miR-558 [31]. CircMAN2B2 was reported to play an oncogenic role in pulmonary cancer via sponging miR-1275 and upregulating downstream FOXK1 [32]. In the current study, high expression level of hsa_circ_0002577 was correlated with poorer overall survival rate, more advanced tumor stage, and severer LNM and LVS in EC patients. The transfection of EC cells with Lv-circRNA promoted growth and decreased apoptosis of EC cells, whereas hsa_circ_0002577 deficiency exerted an opposite effect. The medium collected from EC cells overexpressing hsa_circ_0002577 also induced the formation of tubule-like endothelial structures in HUVECs. Further bioinformatic analysis predicted the potential binding sequence of hsa_circ_0002577 for miR-625-5p. A previous study demonstrated the anti-tumor property of miR-625-5p in gastric cancer [33]. Here, we showed that hsa_circ_0002577 acted as a miR-625-5p sponge that reversely modulated the expression of miR-625-5p in EC cells. Also, the level of miR-625-5p in tumor tissues was significantly lower than that in matched normal samples.

MiR-625-5p was predicted to harbor a potential binding site for IGF1R. A study reported that miR-625-5p targeted IGF1R in colorectal cancer cell line [34]. Consistently, we confirmed that IGF1R was a downstream target of miR-625-5p in EC cells using dual-luciferase reporter assay. IGF1R is a transmembrane tyrosine kinase receptor involved in several intracellular signaling pathways, such as PI3K/Akt and mitogen-activated protein kinase signaling cascades [35]. A significant elevation in IGF1R expression was observed in advanced EC tissues compared to the samples at early stages or proliferative endometrium, indicating that IGF1R overexpression was associated with poorer outcome in EC patients [36]. Therapies targeting IGF1R using IGF1R monoclonal antibody or IGF1R-selective inhibitor are under evaluation for their ability to repress tumor growth and metastasis, and increase the sensitivity of tumor cells to other biological therapies [37]. Consistent with previous findings, the expression of IGF1R in EC tissues was significantly higher than that in matched controls. Moreover, the delivery of IGF1R-overexpressing vectors in cells transfected with sh-circRNA reversed the inhibitory effects of hsa_circ_0002577 deficiency on EC cell proliferation and migration/invasion, suggesting a tumorigenic property of IGF1R in EC progression.

The mutation on *PIK3CA*, a gene encodes a catalytic subunit of PI3K, has been shown as a commonly mutated oncogene in EC [38]. The inhibited phosphorylation of mammalian target of rapamycin, a key downstream mediator of PI3K/Akt pathway, impeded cell proliferation and promoted autophagy in EC cells, suggesting the involvement of PI3K/Akt activation in EC development [39]. In this study, the activation of PI3K/Akt signaling pathway was inhibited in hsa_circ_0002577-deficient cells, but restored to a normal level via excessive production of IGF1R.

## Conclusions

Taken together, this study demonstrated that circRNA hsa_circ_002577 accelerated EC progression by acting as a miR-625-5p sponge, upregulating IGF1R and activating the PI3K/Akt pathway. These findings support the use of hsa_circ_002577 as a potential therapeutic approach in EC treatment.

## Supplementary information

**Additional file 1 Supplementary Figure 1.** The expressions of other upregulated circular RNAs in EC tissues and the Kaplan-Meier survival analysis of EC patients with different miR-625-5p expressions. (A) The expression levels of hsa_circ_0005797, hsa_circ_0057780, and hsa_circ_0016595 in EC vs. normal tissues (*n* = 84). (B) The Kaplan-Meier survival analysis of EC patients with high (*n* = 42) and low (*n* = 42) expressions of miR-625-5p.

## Data Availability

All data generated or analyzed during this study are included in this published article.

## Abbreviations

EC: Endometrial cancer
circRNA: Circular RNA
IGF1R: Insulin-like growth factor 1 receptor
FIGO: Federation of Gynecology and Obstetrics
PI3K: Phosphoinositide 3-kinase
WDR26: WD repeat domain 26
