# Supplementary figures and images for: CircRNA hsa_circ_0002577 accelerates endometrial cancer progression through activating IGF1R/PI3K/Akt pathway
Source: J Exp Clin Cancer Res. 2020 Aug 26;39:169. doi: 10.1186/s13046-020-01679-8 (PMC7450704; doi:10.1186/s13046-020-01679-8)

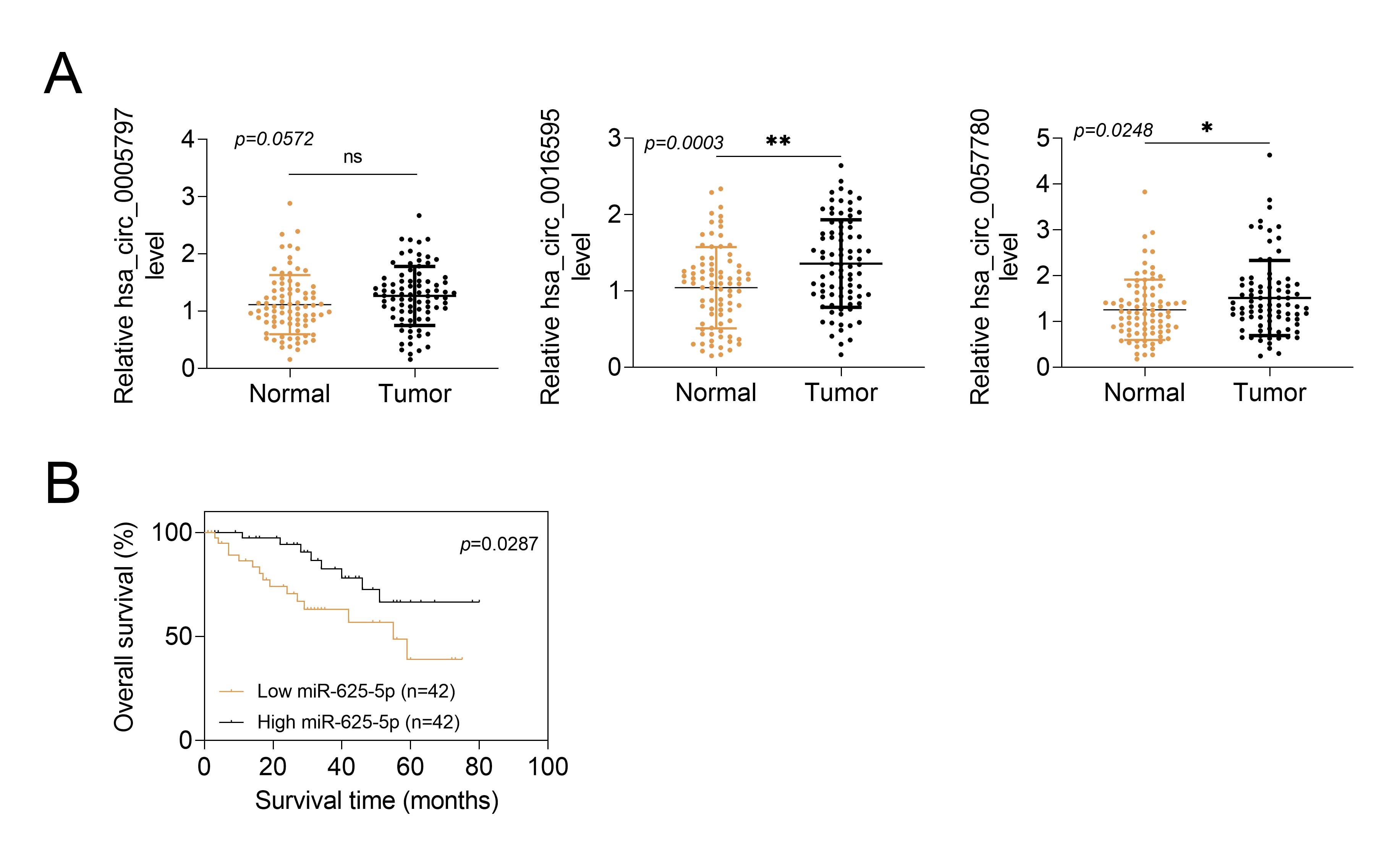

Supplement: Supplementary file 1 — Additional file 1 Supplementary Figure 1. The expressions of other upregulated circular RNAs in EC tissues and the Kaplan-Meier survival analysis of EC patients with different miR-625-5p expressions. (A) The expression levels of hsa_circ_0005797, hsa_circ_0057780, and hsa_circ_0016595 in EC vs. normal tissues (n = 84). (B) The Kaplan-Meier survival analysis of EC patients with high (n = 42) and low (n = 42) expressions of miR-625-5p. [file 13046_2020_1679_MOESM1_ESM.jpg]
